# Considerations for Issues of Regression to the Mean and Contextual Effects in Clinical Trials for Pain in Rheumatic Diseases

**DOI:** 10.1002/acr.25636

**Published:** 2025-09-25

**Authors:** Yen T. Chen, Guohao Zhu, Afton L. Hassett, Daniel Clauw, Susan L. Murphy

**Affiliations:** ^1^ University of Michigan Ann Arbor

## Abstract

Recently, there has been growing discussion about how to best assess pain in clinical trials in rheumatic diseases. Reliable measurement of pain outcomes is essential for accurately determining the effectiveness of treatments. Although pain intensity is the most common measure of change in pain trials, other pain‐related measures, such as pain interference, are also frequently assessed. Interpreting treatment effects on these outcomes can be complicated due to statistical phenomena, particularly regression to the mean and contextual effects. These issues can substantially distort clinical trial findings, potentially leading to inaccurate conclusions about the efficacy of interventions. The present article provides an overview of regression to the mean and contextual effects, emphasizing their implications for internal validity, clinical decision‐making, and ethical considerations in trials. Additionally, this article highlights key study design and analysis considerations, including methodologic and statistical approaches, that researchers can implement to mitigate or better account for these challenges. Practical recommendations are offered to enhance the rigor of pain assessment, with specific attention to osteoarthritis as a representative example within rheumatic disease research. By recognizing and addressing regression to the mean and contextual effects proactively, researchers can strengthen trial outcomes, improve clinical interpretations, and support the identification of effective treatments.

## Introduction

In clinical trials designed to examine treatments for people with rheumatic diseases, pain improvement is often a key objective. However, there are difficulties in designing and interpreting the results of clinical trials when pain is the study outcome. Pain is complex and varies across individuals by intensity, persistence, location, quality, and mechanism (eg, nociceptive, neuropathic, nociplastic). Whereas reviews have discussed considerations for selecting comprehensive pain assessments, including psychosocial factors and related impact on functioning,^1^, ^2^ this article focuses on methodologic concerns that affect pain assessment in clinical trials. In particular, we highlight two critical issues, regression to the mean and contextual effects, both of which can significantly influence trial outcomes. Although these issues are relevant for trials measuring pain intensity, similar considerations may also apply to other pain‐related outcomes, such as pain interference. We describe how regression to the mean and contextual effects arise and outline strategies to better understand and mitigate their impact to improve the interpretation and validity of clinical trial findings.

## Regression to the mean

Regression to the mean refers to the phenomenon in which extreme measurements tend to be closer to the average when measured in subsequent time points.^3^ Among people with chronic pain, such as osteoarthritis (OA), these extreme baseline ratings may reflect temporary pain exacerbation or natural day‐to‐day fluctuations.^3^, ^4^ As a result, even without any treatment, follow‐up pain ratings may appear improved simply because the initial measurement captured an unusually high level of pain. Researchers may be incorporating this bias in trials because they often recruit people with moderate to high pain in their studies so that there is room for participants to improve with treatment. Additionally, potential participants may be more apt to seek out studies when they are experiencing higher levels of pain to get some relief. Having participants who have higher pain than the population of people with OA may be contributing to regression to the mean. Current research guidelines suggest including participants who have a pain rating of at least mild to moderate pain (4 of 10).^5^, ^6^ Although it is important for individuals to have some level of pain to be appropriate for studies investigating pain improvement, the higher the pain threshold for the inclusion criterion, the more the results will be influenced by regression to the mean.^7^ If most participants in a sample have elevated pain ratings compared to their usual pain, there will be an effect of regression to the mean that is not a true treatment effect.^8^ Treatment effects may especially be overestimated due to regression to the mean for within‐group comparisons or in one‐arm trials. This biased treatment effect is due to the natural improvement in a placebo arm that is not accounted for when only one group's data are being analyzed.^3^ In these cases, the minimal clinically important difference (MCID) should be interpreted cautiously.^3^

There are several strategies that can help reduce the effect of regression to the mean (see Table 1). These strategies can be implemented during both the design and analysis phases to improve the validity and interpretability of study findings.

**Table 1:** Considerations for RTM in studies and potential strategies*

Design/analysis	Potential issues	Strategies
Design phase		
Moderate to high pain required for study enrollment	Inflates RTM effects	Use run‐in periods at baseline to reduce measurement variance/error^9^
		Including other coprimary outcome measures^10^ (eg, patient global impression of change^11^ or physical function^12^)
Conducted a one‐arm trial	Overestimates treatment effects	Conduct a randomized trial with a control group to examine natural course of pain over time^8^
Threshold‐based enrollment	RTM amplified at cutoff points	Use a regression‐discontinuity design to compare outcomes for participants just above/below cutoff^14^
Inability to control individual variability	RTM distorts within‐subject changes	Use crossover designs so participants serve as their own controls^15^
		Consider stratified controls to balance baseline pain across arms^16^
Analysis phase		
No analyses to examine RTM	Misinterprets study findings	Plot change scores to visually examine the effect of RTM^17^
		Use ANCOVA to control for individual baseline measure^8^
Limited control for extreme baselines	Exaggerated treatment effects	Apply statistical correction formulas using test‐retest correlations^18^
		Use Bayesian hierarchical modeling to shrink extreme baselines^19^, ^20^, ^21^

* ANCOVA, analysis of covariance; RTM, regression to the mean.

During the designing phase, researchers can consider incorporating a run‐in period with multiple baseline measurements to reduce variability due to short‐term fluctuations in pain.^9^ This approach helps to ensure more stable baseline estimates and to minimize the likelihood that participants are enrolled during a temporary pain flare, which could otherwise result in exaggerated improvements due to regression to the mean. In addition to a run‐in period, researchers can consider including a coprimary outcome measure that is less susceptible to short‐term changes.^10^ Using multiple outcome metrics, such as combining pain intensity ratings with patient global impression of change^11^ or physical function,^12^ provides a more robust assessment of treatment effects and can help verify whether observed changes are consistent or are likely driven by regression to the mean. Regression to the mean seems to be most pronounced for pain intensity, particularly when participants are selected based on moderate to high pain levels, and evidence suggests that other patient‐reported outcomes such as physical function and quality of life may be less susceptible to this effect. For example, Englund and Turkiewicz found that the degree of regression to the mean was relatively small for physical function and quality of life compared to knee pain intensity, likely because trial enrollment typically conditions on pain rather than function or quality‐of‐life scores.^13^ Incorporating a broader range of outcomes may therefore help reduce overinterpretation of changes driven by statistical artifacts. Researchers may also consider incorporating a control group, which is one of the most effective ways to address regression to the mean. By randomizing participants into both intervention and control arms, the effects of regression are distributed across groups, making it easier to isolate true treatment effects.^8^ For studies that use a threshold‐based enrollment criterion, such as including only participants with moderate to high baseline pain levels, researchers can consider a regression‐discontinuity design.^14^ This design compares outcomes for participants just above and below the eligibility cutoff, helping to distinguish intervention effects from regression to the mean.

Design strategies that account for individual variability and extreme baseline scores are also important. Researchers can consider using a crossover design,^15^ in which each participant receives both the intervention and control conditions in separate study periods, with a washout period in between. This within‐subject design allows participants to serve as their own controls, eliminating between‐subject variability and minimizing the influence of regression to the mean, which is expected to occur similarly across both conditions. However, if a participant begins the first study period during a pain flare, the change observed during that period may be more pronounced than that in the second period. This may introduce bias into treatment comparisons. To address this, researchers may consider including repeated pretreatment assessments to better characterize initial symptom levels. Still, crossover designs may not always be feasible, particularly in studies of chronic conditions in which the intervention has a lasting effect or washout periods are impractical. In such cases, researchers can consider using stratified randomization.^16^ Stratification involves grouping participants into subgroups (strata) based on baseline variables before randomization, ensuring that the distribution of baseline scores is balanced across study arms. In addition to improving balance at baseline, stratification also allows for data to be analyzed within each stratum and then combined to estimate an overall treatment effect. However, this approach requires a sufficient sample size within each stratum to ensure more accurate estimates and maintain statistical power, which may be challenging in studies with small samples. When feasible, stratified randomization helps control for baseline imbalances and reduce the distortion of treatment effects that can result from regression to the mean.

In the analysis phase, several statistical approaches can be used to detect or adjust for the effects of regression to the mean. A simple yet informative method is to examine individual or group‐level change scores by plotting pre‐ and postintervention data.^17^ Visual inspection of scatter plots or line graphs displaying baseline versus follow‐up pain intensity ratings can help identify whether improvements are more likely due to natural fluctuations rather than true treatment effects, particularly when participants begin with extreme baseline values. Including these plots provides readers with tangible examples to facilitate understanding and interpretation of potential regression to the mean. A more formal method is to use analysis of covariance (ANCOVA), which includes baseline values as covariates in the model.^8^ This approach statistically adjusts for initial score differences between groups and provides a more accurate estimate of the treatment effect by controlling for baseline variability. ANCOVA is especially useful in randomized controlled trials in which some residual imbalance in baseline values may still occur despite randomization. Researchers may also consider applying statistical correction formulas that adjust for the test‐retest reliability of the outcome measure.^18^ These formulas estimate the amount of change that could be expected due to regression to the mean alone, based on the stability of the measure over time. Incorporating such corrections can help differentiate treatment effects from statistical artifacts and improve the interpretability of study results. Finally, to address the influence of extreme baseline values more robustly, researchers can consider using Bayesian hierarchical modeling.^19^ This analytic technique models individual‐level outcomes as drawn from a broader population distribution and applies a shrinkage effect, pulling extreme values toward the group mean. By reducing the weight of outlier scores, especially those that may be disproportionately affected by regression to the mean, Bayesian hierarchical models provide more stable and reliable estimates of treatment effects. This method is particularly advantageous in studies with small sample sizes or high variability at baseline, in which the risk of regression effects is greater.^20^, ^21^

## Contextual effects

Beyond the direct effects of the intervention itself, patient outcomes are profoundly influenced by contextual effects, nonspecific elements inherent in the clinical or research setting.^22^, ^23^, ^24^, ^25^ These contextual effects, which comprise patient and clinician characteristics, along with psychosocial and environmental factors, can alter the experiences of individuals with OA beyond specific treatment.^26^ They arise from multiple dimensions of the therapeutic encounter, including patient beliefs and expectations, clinician behaviors (eg empathy, communication style),^27^ patient–therapist relationship and/or interactions (eg, trust, nonverbal cues),^28^ and the treatment setting.^29^ For example, patients may report lower pain scores not because of physiologic improvement but because they trust their clinician, feel supported, or interact with clinicians who express optimism about treatment. Research shows these contextual factors, such as provider's warmth, empathy, and communication style, can significantly influence reported pain outcomes in OA.^30^, ^31^ Collectively, these contextual factors can substantially influence treatment outcomes.^26^, ^32^, ^33^, ^34^

These contextual factors are also important to consider because they trigger psycho‐neurobiologic responses within the patient,^25^, ^27^ actively modulating pain perception and leading to changes at neurobiologic, perceptual, and cognitive levels.^25^ Notably, placebo responses can sometimes exceed the effect size of active treatment, highlighting the substantial influence of contextual effects.^24^ Furthermore, contextual effects are not uniformly transient or predictable. Previtali et al found that placebo responses to injections in OA could persist long term and vary across outcome measures, challenging the assumption that these effects are short lived or homogeneous.^35^ Positive contexts (eg, supportive clinician interactions) can enhance placebo effects, improving pain relief, whereas negative contexts (eg, distrust or pessimism) may induce nocebo effects, worsening symptoms.^25^

Understanding how contextual effects impact pain perception is crucial for accurately interpreting interventions and optimizing clinical care in OA and other rheumatic diseases.^25^, ^36^, ^37^ In randomized controlled trials (RCTs), the primary goal has traditionally been to isolate and quantify the treatment efficacy. This is typically done by comparing the outcomes in an active intervention group to those in a placebo or sham control group. The placebo arm is intended to capture nonspecific effects, allowing researchers to subtract them and determine the effect solely attributable to the active intervention. However, recent research has highlighted that contextual effects present in both the active treatment arm and placebo or sham arm contribute significantly to the observed outcomes in RCTs.^38^ Studies on nonpharmacological interventions, such as exercise and acupuncture, indicate that contextual effects, including placebo effects and natural symptom fluctuations, play a dominant role in perceived pain relief in patients with knee OA.^26^ A meta‐analysis of OA RCTs found that, on average, 75% of the observed pain reduction was attributable to the contextual effects.^39^ The proportion attributable to contextual effect (PCE) can vary depending on the specific treatment. For instance, in OA pain reduction, PCE ranged from 47% for intra‐articular glucocorticoids to 91% for joint lavage.^39^

The high proportion of benefit attributable to context gives rise to what is known as the “efficacy paradox.” Although RCTs are designed to isolate and test specific treatment effects (which are often modest or below clinically meaningful thresholds), real‐world clinical outcomes reflect the combined impact of specific and contextual effects.^40^ Because contextual effects are always present in clinical encounters and can contribute substantially to the overall response,^32^ a treatment with a small specific effect might still be perceived as clinically beneficial by patients and clinicians.^39^, ^41^ For example, Saueressig et al note that the estimated contextual effect for pain intensity is 5.3 on a 0‐ to 100‐point visual analog scale, which makes up more than 25% of the typical MCID for pain (20 points) and 30% to 40% of the effect of commonly recommended treatments such as exercise, psychosocial interventions, and multidisciplinary management.^42^ This highlights that the context is a sizable component that should not be disregarded.

The efficacy paradox complicates the interpretation of RCT results. A statistically significant difference between an active treatment and a placebo may reflect only a small specific effect, overshadowed by a large contextual effect present in both groups. Conversely, the absence of a significant difference does not necessarily mean that the total treatment package (comprising both the specific and contextual effects) is ineffective in clinical practice. Thus, focusing solely on the specific effect measured by the difference between active and placebo arms can obscure the substantial benefits that patients may derive from the contextual aspects of care.^39^, ^40^ Although enhancing contextual factors has been proposed as a cost‐effective strategy to improve outcomes, one meta‐analysis suggested that intentionally boosting contextual effects alone in nonpharmacological treatments may offer limited additional clinical benefit.^42^ Nevertheless, this does not diminish the essential role contextual factors play in standard care. Contextual effects should be viewed as an integral component of treatment outcomes, not merely as confounding elements to be minimized or subtracted.^43^

To better interpret treatment effects in pain studies for OA and other rheumatic diseases, researchers must actively account for contextual effects. If unaddressed, contextual effects can bias estimates of treatment efficacy and lead to misleading conclusions. A comprehensive strategy to reduce and interpret contextual effects should involve both study design and measurement and analytic approach (see Table 2).

**Table 2:** Considerations for contextual effects and potential strategies*

Design/analysis	Potential issues	Strategies
Design phase		
One‐arm or two‐arm RCT (active vs placebo)	Difficult to isolate contextual effects from treatment effects	Use three‐arm RCT to add untreated control to quantify contextual effects (placebo vs untreated)^44^
		Use cluster randomization to control for provider‐level biases^45^
Unstandardized patient interactions	Variability in placebo/nocebo responses due to uncontrolled expectations	Use standardized scripts to balance explanations of benefits/risks to minimize bias^22^
		Include active placebos to mimic side effects^48^
		Use placebo run‐in phase to exclude high responders^49^
Clinician/interventionist variability (eg, empathy, communication)	Placebo effects or nocebo effects from positive or negative interactions	Use blinded outcome assessment (independent evaluators)^50^
		Have standardized protocol and treatment environment^51^
Inability to disentangle expectancy vs treatment	Biased efficacy estimates	Use balanced placebo designs/open‐hidden paradigms^52^
		Use crossover/randomized withdrawal designs^54^
No qualitative data	Missed insights into patient experiences	Include qualitative data to explain quantitative results^55^, ^56^
Measurement and analysis approach		
Lack of psychosocial measures	Confounding by unmeasured mood/stress/social support	Use expectation rating scales (eg, Credibility/Expectancy Questionnaire)^57^
		Include validated scales (eg, PROMIS depression, PROMIS anxiety, social support) as covariates/mediators^36^, ^59^
Ignoring proportional contextual effect	Overemphasis on specific effects, undervaluing contextual benefits	Report proportional contextual effect alongside specific/overall effects^39^, ^40^

* PROMIS, Patient‐Reported Outcomes Measurement Information System; RCT, randomized controlled trial.

A core design recommendation is the use of a three‐arm RCT design that includes an active intervention group, a placebo or sham group, and a nontreated control group.^44^ This design allows researchers to estimate the contextual effects by comparing outcomes between the placebo and untreated groups while isolating the specific treatment effect through comparison of the active and placebo groups.^29^ This structure also helps adjust for symptom fluctuations and regression to the mean. To further control for provider‐related contextual effects, researchers can implement cluster randomization,^45^ in which participants are assigned by treatment provider. This approach reduces variability caused by individual differences in clinician behavior or communication. However, when there are a small number of clusters or systematic differences in provider characteristics across study arms (eg, if providers in one arm tend to have better communication skills), cluster randomization may actually increase the risk of biased contextual effects. There are other trade‐offs to cluster randomization that have been outlined in several reviews.^45^, ^46^, ^47^

Additionally, standardizing participant–provider interactions is also critical in reducing contextual effects. Researchers and research personnel should use scripts that provide a consistent explanation of potential treatment benefits and risks. This helps ensure that expectations are managed similarly across study arms. Training health care providers to create a positive therapeutic environment and manage patient expectations can harness the beneficial components of contextual effects while minimizing their confounding influence on the measured efficacy of active treatment.^22^ This approach not only improves clinical outcomes but also aligns with a more holistic understanding of pain management.^23^ Maintaining blinding of those delivering the intervention is another crucial strategy. The use of active placebos that mimic side effects of the intervention can equalize perceived credibility of treatment arms.^48^ Researchers may also consider a placebo run‐in phase,^49^ which allows for identification and exclusion of individuals who show strong placebo responses before randomization, thereby enhancing the precision of treatment effect estimates.

To reduce bias in outcome measurement, clinical trials should use independent evaluators who are blinded to participants’ treatment assignments.^50^ Blinding helps make sure that assessments are not influenced by conscious or unconscious expectations about the efficacy of the intervention. This is especially important in trials involving subjective outcomes, such as self‐reported pain, in which evaluator interactions or assumptions could unintentionally shape the data collection process. In addition to blinding, maintaining a standardized treatment environment across all study arms is critical.^51^ This includes using consistent physical settings, materials, schedules, and procedures so that participants across groups are exposed to similar contextual cues. Standardization helps prevent differences in the care environment from contributing to variability in outcomes, allowing for a more accurate estimation of the treatment's true effect.

For trials specifically aiming to disentangle the effects of patient expectations from the active components of treatment, researchers can use advanced experimental designs such as balanced placebo designs and open‐hidden paradigms.^52^ These approaches manipulate what participants are told about the treatment to separate expectancy effects from the pharmacological or procedural components. For example, in an open‐hidden paradigm, patients might receive an analgesic either openly (with full awareness and explanation from a clinician) or hidden (administered without their knowledge). Research using this design has shown that the same medication can produce stronger pain relief when patients are aware they are receiving it, compared to when it is administered covertly, highlighting the influence of expectations on treatment response. This method has been used in both pharmacological and nonpharmacological pain research to better understand how much of the benefit is attributable to the treatment itself versus the patient's belief in its effectiveness.^33^, ^53^ In addition, crossover or randomized withdrawal designs can help evaluate whether observed effects persist or reverse after the intervention is discontinued,^54^ providing further insight into the durability and origin of contextual effects.

Finally, because contextual effects often involve subjective experiences, qualitative research is an important complement to quantitative designs. Interviews and open‐ended feedback from participants can provide insight into how individuals perceive the intervention and what they believe contributed to their outcomes.^55^, ^56^ By incorporating qualitative assessment into quantitative studies or using mixed‐methods designs, researchers can better account for the contextual effects of pain, leading to more accurate interpretations of treatment efficacy in pain studies.

Beyond study design, measurement and analytic strategies also play a central role in accounting for contextual effects. One important step is to measure patient expectations at baseline using validated tools such as the Credibility/Expectancy Questionnaire.^57^ These scores can later be analyzed to determine whether expectations mediate or moderate treatment outcomes.^58^ Other psychosocial variables known to influence pain, such as mood, anxiety, and social support, should also be systematically assessed. Validated instruments such as the Patient‐Reported Outcomes Measurement Information System depression and anxiety scales and social support measures can be included as covariates in statistical models.^36^, ^59^ Their inclusion allows researchers to adjust for or explore the modifying effects of these psychosocial factors on treatment outcomes. In studies in which the goal is to examine mechanisms, causal mediation analysis can be used to determine whether these contextual variables account for part of the observed treatment effect.^36^

To improve the interpretation of RCT results, researchers are strongly encouraged to report not only the traditional specific treatment effect (difference between active and placebo) but also the overall treatment effect (improvement in the active group) and the PCE.^39^, ^40^ This approach provides a more complete, balanced, and clinically meaningful interpretation. Adhering to reporting guidelines for contextual factors, such as the Control Interventions in Efficacy Trials of Physical, Psychological, and Self‐Management Therapies statement,^60^ is also advised.^32^

## Conclusions

In clinical trials that evaluate pain, two common sources of issues, regression to the mean and contextual effects, can lead to problems in misunderstanding study findings and should be considered in research. This article emphasizes the importance of addressing these issues during both the design and analysis phases of pain research. Strategies such as incorporating three‐arm RCTs including run‐in periods, collecting data on psychosocial variables, applying appropriate statistical adjustments, and integrating qualitative methods can strengthen the validity of study findings. These strategies provide a more complete understanding of how both specific and nonspecific factors influence outcomes, leading to more accurate and reliable conclusions. By doing so, researchers can design trials that better reflect the complexity of real‐world care and contribute to the development of more effective and meaningful interventions for patients.

## AUTHOR CONTRIBUTIONS

All authors contributed to at least one of the following manuscript preparation roles: conceptualization AND/OR methodology, software, investigation, formal analysis, data curation, visualization, and validation AND drafting or reviewing/editing the final draft. As corresponding author, Dr Chen confirms that all authors have provided the final approval of the version to be published and takes responsibility for the affirmations regarding article submission (eg, not under consideration by another journal), the integrity of the data presented, and the statements regarding compliance with institutional review board/Declaration of Helsinki requirements.

## Supporting information


**Disclosure Form**:
